# P004 Progressive insufficiency fractures and structural foot collapse associated with methotrexate: a case report with evidence of re-challenge and de-challenge effect

**DOI:** 10.1093/rap/rkag113.034

**Published:** 2026-11-03

**Authors:** Heba Fahmy, Gillian Roberts

**Affiliations:** Inverclyde Royal Hospital, Glasgow, United Kingdom; Inverclyde Royal Hospital, Glasgow, United Kingdom

## Abstract

**Introduction:**

Methotrexate (MTX) is widely used in the management of inflammatory rheumatological conditions. Although generally well tolerated, rare complications such as methotrexate-associated osteopathy (MTXO) have been increasingly reported. This entity is characterised by insufficiency fractures, most commonly affecting the lower limbs, and may mimic inflammatory or mechanical pathology, leading to delays in diagnosis. Early recognition and prompt withdrawal of MTX are essential to prevent progressive skeletal damage and irreversible structural complications.

**Case description:**

A 60-year-old woman with anti-NXP2 positive dermatomyositis diagnosed in 2023 was commenced on oral methotrexate in June 2023, later switched to subcutaneous administration in September 2023. Her past medical history included coronary artery bypass grafting and mild left ventricular systolic dysfunction.

MTX was discontinued in April 2024 due to mucositis and alopecia.

Shortly thereafter, she developed severe left ankle pain associated with significant functional limitation, requiring two walking aids. The clinical picture was atypical, with minimal swelling and features not consistent with inflammatory arthritis. Plain radiographs were unremarkable.

Magnetic resonance imaging (MRI) of the left ankle performed in May 2024 demonstrated an incomplete fracture involving the posterior facet of the subtalar joint with associated bone marrow oedema, in the context of underlying osteopenia.

Despite these findings, methotrexate was reintroduced at 20 mg weekly in July 2024 and subsequently increased to 25 mg weekly in November 2024.

Following re-exposure, the patient experienced progressive symptoms, with involvement of both feet and multiple joints. Repeat MRI of both ankles in February 2025 demonstrated multiple insufficiency fractures, including the navicular, medial cuneiform, and calcaneus, with extensive bone marrow oedema.

MTX was discontinued again in April 2025.

A follow-up MRI performed five months later demonstrated interval improvement in bone marrow oedema and partial healing of stress fractures. However, there was evidence of structural damage, including loss of navicular bone stock and features suggestive of developing Mueller-Weiss syndrome.

**Discussion:**

MTXO is an increasingly recognised but likely underdiagnosed complication of long-term methotrexate therapy. Recent case series, predominantly involving patients with rheumatoid arthritis, have described characteristic lower limb insufficiency fractures and highlighted the risk of delayed diagnosis due to overlap with inflammatory or mechanical symptoms.

Our case demonstrates that this complication can also occur in anti-NXP2 dermatomyositis, expanding the spectrum of rheumatic diseases in which methotrexate osteopathy should be considered. The observed dechallenge–rechallenge phenomenon, with radiological progression following methotrexate reintroduction and improvement after withdrawal, provides strong evidence supporting a causal relationship. Despite partial healing, persistent structural collapse emphasises that delayed recognition may result in irreversible skeletal damage.

Clinicians should maintain a high index of suspicion in patients receiving methotrexate who develop unexplained lower limb pain, even when initial radiographs are normal, and MRI should be considered early to facilitate diagnosis and prevent permanent disability.

**Key learning points:**

• MTXO is an uncommon but increasingly recognised cause of insufficiency fractures in patients receiving long-term MTX. It should be considered in patients presenting with persistent or atypical lower limb pain, particularly when symptoms are disproportionate to clinical findings and plain radiographs are normal.MRI is valuable for early diagnosis. This case provides evidence of both a de-challenge and re-challenge effect, with progression of fractures following MTX reintroduction and radiological improvement after withdrawal, strengthening the causal association. Early recognition and prompt discontinuation of methotrexate may prevent progressive fractures, irreversible structural damage, and long-term disability.

